# Role perception of infection control link nurses; a multi-centre qualitative study

**DOI:** 10.1177/17571774211066786

**Published:** 2022-02-18

**Authors:** Mireille Dekker, Rosa van Mansfeld, Christina MJE Vandenbroucke-Grauls, Tessa E Lauret, Bernadette CFM Schutijser, Martine C de Bruijne, Irene P Jongerden

**Affiliations:** 1 Department of Medical Microbiology and Infection Prevention, Vrije Universiteit Amsterdam, 1209 Amsterdam UMC, Amsterdam, The Netherlands; 2 Department of Public and Occupational Health, Amsterdam Public Health research institute, Vrije Universiteit Amsterdam, 1209 Amsterdam UMC, Amsterdam, The Netherlands

**Keywords:** Infection prevention, compliance, safety II, liaison, qualitative research

## Abstract

**Background:**

Infection control link nurses (ICLN) disseminate knowledge on infection prevention topics to their peers. Little is known about how they succeed and thereby contribute to infection prevention in daily practise.

**Aim:**

To explore the experiences of infection control link nurses regarding their role in acute care hospitals and identify perceived facilitators and best practices.

**Methods:**

We conducted a qualitative study with semi-structured individual and focus group interviews with ICLN. The effect of COVID-19 on the ICLN role was added as a topic in focus group interviews during the pandemic.

**Results:**

Twenty-six ICLN working in acute care hospitals were interviewed. ICLN perceived their role as to identify, monitor, facilitate and inform their colleagues on infection prevention topics related to their ward. Their experiences vary from feeling challenged and wonder how to get started, to feeling confident and taking initiatives that lead to ward-based improvements. When inspired by each other and supported by infection control practitioners or managers, ICLN feel empowered to initiate more activities to improve practice. During the COVID-19 pandemic, ICLN felt their responsibilities were magnified. When transferred to another ward, the focus on the ICLN role seemed dispersed.

**Discussion:**

Empowered ICLN adjust and operationalize infection prevention policies to fit the conditions of their specific wards and provide practical instructions and feedback to their peers which enable better compliance to infection prevention policies. Support and inspiration from other ICLN, infection control practitioners and management contribute to this empowerment and consequently to taking impactful initiatives to improve practice.

## Background

Infection control link nurses (ICLN) are role models in providing safe care and transfer their knowledge and skills to their peers (Dawson, 2003). In order to fulfil this role, link nurses in acute care hospitals are trained by infection control practitioners. Programs to support ICLN vary in the way they are organized from occasional education to well-designed programs that also provide training in implementation skills through train-the-trainer principles (Dekker et al., 2019, 2020). Implementation of the link nurse role depends on local priorities; ad hoc practice is common (Dekker et al., 2019, 2020).Prior studies have mainly focused on ICLN roles from an organizational perspective (Dekker et al., 2019; Peter et al., 2018). Little is known about the way link nurses themselves perceive their role; how they fulfil it, how they increase and disseminate their knowledge, what difficulties they encounter, and what supports them in advocating infection prevention in clinical practice. The few studies that have assessed the ICLN perspective, focused on the ICLN profile with accompanying roles and tasks and on educational strategies (Cooper, 2005; Teare et al., 2001; Williams et al., 2019). In a qualitative study, experiences of 10 ICLN with a 6 month ICLN program were evaluated, revealing self-reported empowerment and self-reported improvement of clinical practice (Cooper, 2005). Other papers provided suggestions for the education of link nurses, mechanisms to support them and the legitimation of the role (Williams et al., 2019; Teare et al., 2001). Although these aspects deserve attention, they fail to help in understanding how ICLN endeavour to disseminate their knowledge and improve practice, and what hinders and facilitates them during their activities. Examining these issues could provide better insight in how ICLN contribute to the improvement of infection prevention at the ward level and how ICLN programs could optimally facilitate these contributions. We therefore sought to explore the experiences with and perceptions of ICLN regarding their role in acute care hospitals.

## Methods

### Study design

Between April 2019 and December 2020, we conducted a qualitative study in which we combined face-to-face semi-structured interviews and online focus group interviews with ICLN from five Dutch hospitals. We followed the Consolidated Criteria for Reporting Qualitative Research (Tong et al., 2007).

### Participant selection

To maximize variation in perspectives, we recruited ICLN from inpatient wards and outpatient clinics from three university hospitals and two general hospitals with varying ICLN programs. All ICLN practicing in inpatient and outpatient settings were eligible to participate. They were invited to participate by email by the hospital’s infection control practitioner. Twenty three ICLN responded and received an information letter about the aim and procedure of the study and the voluntary nature of the study.

### Data collection

Semi-structured face-to-face interviews were performed to capture and understand personal views, opinions and experiences (Moser and Korstjens, 2018). These interviews were conducted by three female researchers (MD, BS and TL) trained in qualitative methodologies and interview techniques. MD is an infection control practitioner and a clinical epidemiologist, TL is an infection control practitioner and BS is a fulltime researcher with a nursing background. Interviews took place between April 2 and 25 June 2019 at a convenient time in a private room at the participants’ hospital. The researchers did not know the participants they interviewed.

An interview guide (Table 1) based on recent literature on ICLN was used (Dekker et al., 2019, 2020). The interviews started with asking the ICLN to describe their role in general and to provide examples of their activities as an ICLN. Follow-up questions encouraged them to express their thoughts and perceptions more thoroughly. The interviews lasted between 29 and 54 min and were audio-recorded with the consent of the interviewees. Field notes on the interviews were documented by the interviewers directly after each interview. After 15 interviews, no new subjects came forward. We planned two extra interviews for checking data saturation, and no new themes emerged. Therefore, after 17 interviews, data saturation was considered to have been reached (Moser and Korstjens, 2018).

**Table 1.** Topic list.

*How did you take on the link nurse role?*
Did you volunteer for the role or were you appointed?
What was the reason for signing up?
*How would you describe the link nurse role?*
How will others know that you are an ICLN?
Can you describe some recent link nurse activities?
Did these activities change over time?
What did change? And why?
What are plans for the future?
Would you have done things in a different way?
What would you have done different?
What would you need for that to do so?
*Did you have to learn to be an ICLN?*
Can you explain that?
How did you know what you had to learn?
Can you relate that to a moment, event or feeling?
*What would help you to fulfil your link nurse role?*
What would be needed for that?
What if these needs cannot be fulfilled?
*Did the current COVID-19 pandemic affect the link nurse role?*
Can you explain that/describe your experiences?
Can you describe how it affected your link nurse activities?

During the course of this study, at the stage of data analysis, the COVID-19 pandemic evolved. We hypothesized that this exceptional situation could have influenced ICLN’ perceptions on their role. Therefore, we included a topic related to the role of ICLN during the first wave of the recent COVID-19 pandemic and performed focus group interviews.

Focus group interviews were conducted using a digital platform (https://zoom.us/) in November and December 2020 and consisted of two to four participants. Three participants were not able to log in for the online focus group interviews due to technical problems (one participant) or patient care duties (two participants). A moderator (MD) led the discussion. An observer (IJ and JJ) took notes on striking topics or non-verbal communication and interaction. The researchers had no formal hierarchical relationship with the participants. Focus group interviews lasted between 42 and 65 min.

### Data analysis

The face-to-face interviews were transcribed verbatim by an independent professional transcriber, checked for accuracy by one researcher (MD) and analysed using thematic analysis (Braun et al., 2019). The focus group interviews were transcribed by one researcher (MD). Two researchers independently (MD and TL) read the transcripts several times to familiarize with the data. The first eight interviews were independently coded by the two researchers by highlighting segments of text in the transcripts and coding these inductively. Differences in the interpretation of text segments or codes were discussed. As consensus was high, the remaining interviews were coded by one researcher (MD) and subsequently discussed by the research team (MD, RM, TL, BS and IJ). An audit trail, consisting of field notes on interviews, memos created during the coding process and annotations of research related discussions, helped to maintain awareness of the teams’ preconceptions and how they could affect the interpretation of findings.

Initial codes were sorted and grouped into categories by one researcher (MD). Subsequent discussions with the research group (MD, RM, TL, BS and IJ) aimed to refine categories and define overarching themes. From the initial 1305 codes, we created 36 categories and three overarching themes. The analysis of the focus group interviews revealed three additional codes. Themes, categories and codes were again reviewed by MD, RM and IJ to improve the quality of the analysis. One researcher (MD) further refined the themes and described the content.

All data was analysed in Atlas.Ti software version 8.0 for Windows.

## Results

Twenty-six link nurses were interviewed: 17 link nurses from five hospitals through individual, face-to-face interviews, and nine link nurses from four hospitals through four online focus groups (Tables 2 and 3).

**Table 2.** Characteristics of link nurses in face-to-face interviews.

	Infection control link nurses (*n* = 17) *n* (%)
Gender	
Female	16 (94)
Setting	
University hospital	13 (76)
General hospital	4 (24)
Department	
Inpatient wards	12 (70)
Outpatient clinics	4 (24)
Diagnostic department	1 (6)
Number of years of experience as a nurse	
6–10	3 (18)
>10	13 (76)
Missing	1 (6)
Number of years of experience as a link nurse	
0–5	10 (59)
6–10	5 (29)
Missing	2 (12)
Position	
Senior nurse	8 (47)

**Table 3.** Characteristics of link nurses in focus group interviews.

	Infection control link nurses (*n* = 9) *n* (%)
Gender	
Female	7 (78)
Setting	
University hospital	8 (89)
General hospital	1 (11)
Department	
Inpatient wards	5 (56)
Outpatient clinics	3 (33)
Diagnostic department	1 (11)

Link nurses volunteered for the role based on their interest in infection prevention or became a link nurse as a part of their position as a senior nurse. In general, link nurses confirmed being interested in the topic and were keen on increasing their knowledge on infection prevention, for themselves and for their colleagues. This interest was based on their motivation to provide safe care or was driven by more personal reasons (e.g. being found to be a carrier of methicillin-resistant *Staphylococcus aureus* during a contact tracing procedure).

The analysis of the interviews led to three main themes: Focus on infection prevention activities in the own ward, improvement by small increments and need for inspiration and support.

### Focus on infection prevention activities in the own ward

ICLN described their role as to identify, monitor, facilitate and inform on infection prevention topics related to their ward. ICLN described observing their colleagues during the provision of care. When non-adherence was noted, some link nurses discussed their observations in one-on-one conversations with their peers. Others discussed their observations in a more general way, during team meetings or described their observations and provided suggestions for improvement in emails or newsletters. In addition to these observations in daily practice, some ICLN performed audits and discussed the results with their colleagues.

I have conversations with my colleagues about the way they provide their care. It gives me an understanding of their knowledge and provided an opportunity to answer questions. I often notice a lack of knowledge. With these conversations I can inform them. [interview 5, university hospital, inpatient ward]

When infection prevention questions on specific patients arose, ICLN acted as an intermediate between their direct colleagues and the infection prevention team. ICLN narrated that they were able to either immediately answer the question, were rapidly able to find the appropriate protocol, or contacted the infection control helpdesk to help their peers to quickly find the answer. ICLN translated infection control policies into explicit work instructions or provided practical solutions to support the applicability of these protocols in situations specific to their ward. Translation of these protocols was done at the initiative of the ICLN or as a response to questions raised by team members. When alleged inconsistencies or infeasibilities in the protocols were found, ICLN did not hesitate to consult the infection control practitioner.

Sometimes, I find infection prevention difficult too, and sometimes I have my doubts. Do we have to disinfect our hands or not? In these situations, I will perform the procedure myself, think it through for a moment, and then report my findings to my peers. [interview 7, university hospital, inpatient ward]

I wrote a cleaning plan for the department. There are quite a few protocols on cleaning and they are long. I extracted the information that is important for my department and to turned it into a plan specific for our department. [interview 2, university hospital, outpatient clinic]

My colleagues found it difficult to assess if they had donned their personal protective equipment in the right way. I arranged a large mirror. [focus group 2, university hospital, inpatient ward]

### Improvement by small increments

ICLN stressed that improvement was only possible with small increments and found that when they brought their information in a fun way it was more likely to stick.

At first my colleagues were reluctant. “Oh no, here we go again, we have to adjust our approach…again.” And now, they start to understand the point of these adjustments. [interview 12, general hospital, inpatient ward]

Some ICLN described the link nurse role as challenging; they did not know where to start, what issues to address or how to outline their activities. These link nurses stated the need for more guidance.

At first, I thought I had to gain knowledge and I would subsequently start to promote infection prevention. Then, I decided to just start some activities. Two weeks ago I promoted the 5 moments of hand hygiene; practice has not changed. I don’t know what to do next. [interview 1, university hospital, outpatient clinic]

Some ICLN reported dealing with resistance of colleagues in the compliance with infection prevention policies. Humour was mentioned as an icebreaker. Self-confidence of ICLN emerged from positive experiences with implementing infection prevention policies, speaking up and addressing colleagues to non-compliance with infection prevention guidelines. It facilitated a pro-active attitude. Self-confidence was perceived as a prerequisite for leading by example and sustained motivation for the role. ICLN were proud of their success in improving safe care and mentioned the incorporation of their link nurse activities into their everyday practice.

Initially, I did not dare to speak up. However, as an ICLN I felt supported by the organization. I became more certain of myself. I started to think differently “I do not speak up for myself, I speak up for the safety of our patients.” Most colleagues had no idea that they did not provide safe care. And well, that of course motivates to speak-up the next time it seems necessary. [interview 5, university hospital, inpatient ward]

Only a few ICLN mentioned that they led by example and that being a role model was an important part of their role.

I see myself as a role model. I know the protocols and I’m also aware of our weaknesses, especially when the workload is high. I am not perfect either. I share and discuss my own flaws with my colleagues and my intentions to do better next time. [interview 5, university hospital, inpatient ward]

### Need for inspiration and support

ICLN described the need for inspiration and support from their peers, the ward management, the infection control practitioner and other link nurses.

#### Inspiration

Educational sessions were mentioned as a source of inspiration to assume the link nurse role. Infection control practitioners provided tools to help ICLN to transfer their knowledge to their peers. Especially discussing their experiences and sharing success stories during these sessions inspired ICLN to apply these strategies in their own ward. Beside educational sessions, ICLN relied on professional literature, protocols and collaborations with the infection control practitioner as sources of knowledge.

The infection control practitioner provides a range of tools to get you started. [interview 10, university hospital, outpatient clinic]

#### Sparring partners

Link nurses stressed the importance of a buddy on the ward to discuss how to execute plans and initiatives. Most link nurses choose a peer as their sparring partner, some wards formally appointed a second ICLN for this purpose.

I have a link nurse buddy. There are many colleagues in my team with a variety of competences that are willing to help. So if I need a sounding board, I can always have a discussion with my buddy or with one of my other colleagues. [interview 2, university hospital, outpatient clinic]

#### Support from the infection control practitioner

A pro-active role was expected from the infection control practitioner and link nurses expressed the availability and accessibility of an infection control practitioner as a precondition to fulfil their role. Infection control practitioners acted as a hotline, a source of information for ad hoc questions and as a coach during more complex questions. Support from the infection control practitioner helped ICLN to operationalize protocols and translate them into workable instructions for their specific department or workflow. ICLN expressed the urge to team up as equal partners. When this support was not readily available, ICLN felt hindered in the execution of their role and questioned the importance of their initiatives.

I’m in close contact with the infection control practitioner. I told her that we needed to organize some education on COVID-19 and the accompanying infection prevention measures. Colleagues did not understand the need of social distancing during coffee breaks, because at the bedside nurses work so closely together. [focus group 1, university hospital, inpatient ward]

#### Support from the ward manager

Link nurses expected their ward manager to acknowledge and validate the link nurse role to the rest of the team, for example, when peers resist to comply with infection control policies. Link nurses felt their role was undermined when this support was not in place.

I know exactly which colleagues do and do not comply. And when I observe non-compliance, I discuss my observations with them. If these conversations have no effect, I can turn to my supervisor. She has much more authority than I do. [interview 8, university hospital, inpatient ward]

#### Collaboration with other link nurses

Most link nurses expressed the need to collaborate with link nurses throughout the hospital, though they did not take any initiative to organize such collaboration.

I would like to see the other ICLN more often; to exchange information and strategies. To learn from each other and to collaborate. [interview 13, general hospital, inpatient ward]

### ICLN in times of the COVID-19 pandemic

During the recent COVID-19 pandemic, ICLN felt their link nurse responsibilities were magnified. Although overwhelmed by the situation and the rapidly changing policies, ICLN felt responsible to read the daily updated COVID-19 protocols and to provide their peers with concise and up-to-date information. ICLN felt their knowledge on infection control contributed to their understanding of the measures and hence their ability to answer questions from their peers.

As a link nurse I had more knowledge on this topic. My colleagues turned to me for answers. There were a lot of questions and a lot of uncertainties. I read the updates on the protocol, sometimes two or three times a day. They expected me to be up-to-date, but also understood that I did not have all the answers either. [focus group 1, university hospital, inpatient ward]

Some ICLN were transferred to another other ward for a short period of time during the first wave of the COVID-19 pandemic. They described that the focus on their link nurse role diminished.

During the first COVD wave I was transferred to the intensive care unit. I thought about the measures and whether they made sense to me, but I kept a low profile… Me too, I was overwhelmed. The infection control department was in control of the donning and doffing policies. I came to support the intensive care nurses; the link nurse role was never discussed at all. It never came to my mind either. [focus group 2, university hospitals, inpatient wards]

## Discussion

In this qualitative multi-centre study, we explored the experiences with and perceptions of ICLN on their role in acute care hospitals. ICLN mainly focus on infection prevention activities in their own ward and seem to restrict their focus on one or two infection prevention topics (e.g. hand hygiene, isolation precautions, cleaning and disinfection policies). ICLN improve practice by small increments as they operationalize infection prevention policies into workable instructions, share their knowledge with peers by answering their questions and observe them during care procedures. The experiences of ICLN with their role vary from feeling challenged to get started to confident initiatives that smoothly lead to ward-based improvements. The perception of ICLN is influenced by positive experiences with their link nurse activities. ICLN are inspired to initiate activities by sharing best practices with other ICLN, bolstered by a pro-active infection control practitioner and support of the ward manager.

Our findings on ICLN’ needs for support from various stakeholders builds on the work of Williams and colleagues, who found that ICLN should have access to formal and informal support mechanisms (Williams et al., 2019). The appropriate operationalization of this support is needed to facilitate ICLN to undertake the role (Bunce et al., 2020; Williams et al., 2019). Therefore, the roles and responsibilities of the ICLN, the team manager, buddies and the infection control practitioner must be defined and balanced at the ward level, with respect to the local culture and power dynamics. If these stakeholders can join forces, conditions are created for effective implementation of safe practices with interventions that are adjusted to local priorities, ward culture and its context-specific facilitators and barriers (Caris et al., 2017; Damschroder et al., 2009; Williams et al., 2016; Zingg et al., 2015). The ward manager has formal authority and is therefore pre-eminently able to affirm the importance of infection prevention and the link nurse role, to provide back-up and strengthen the influence of the ICLN (Bonawitz et al., 2020; McAlearney et al., 2021). Collaboration with peers can help ICLN to overcome resistance and engage team members in improving practice (Bonawitz et al., 2020). The infection control practitioner can facilitate this micro network by providing and translating knowledge on infection prevention. When infection control practitioners also focus on the development of positive relationships with these local micro networks, this facilitates interaction, mutual understanding and therefore enhances adoption of knowledge (Bornbaum et al., 2015). In addition, infection control practitioners can align ICLN from departments that work on similar projects. This way infection control practitioners can provide reliable information and control the application of this information during the planning of these projects (Burt, 2001). This so-called brokerage is known to provide an efficient way of using resources and enhances the ability for ICLN to learn and to collaborate (Bornbaum et al., 2015). The need of interviewees to collaborate with ICLN from other wards is consistent with findings from a study of Hasson et al. in which palliative care link nurses stipulated the need of reinforcement from their link nurse partners (Hasson et al., 2008). Current ICLN programs mainly focus on the transfer of knowledge and skills (Dekker et al., 2020). However, education as a self-contained intervention is known to sort little effect (Grol and Grimshaw, 2003; Soong and Shojania, 2020). This explains why ICLN are only loosely connected and do not take the initiative to organize collaborations (Granovetter, 1973; Putnam, 2000). It could also explain why the link nurse role seems to be bound by the link nurse’s work environment. At the hospital level, this could mean that future ICLN programs should facilitate ICLN to connect within a network that facilitates information sharing, fosters relationships and promotes interdepartmental collaborations. Networks with these features are considered to positively impact implementation and are associated with sustainability and the creative solving of problems (Neal, 2015; Watts and Strogatz, 1998). The ability of ICLN to adopt infection prevention protocols, monitor their compliance and adjust them to fit the unpredictable and complex clinical conditions of their specific wards, aligns with the concepts of the Safety II perspective on healthcare. Safety II facilitates a positive approach with the health care worker at the centre that accepts variation, embraces variability in protocols and encourages flexible ways of working (Hollnagel et al., 2015; Smith and Plunkett, 2019). It can be used to understand the complex processes of the daily practices and sees humans as a part of the solution. The rationale behind it is that protocols and procedures can never anticipate all situations that can occur (Rankin et al., 2014). ICLN that successfully contribute to this flexible way of applying infection prevention and enable their peers to mindfully adapt their care can be defined as resilient or empowered health care workers (Braithwaite et al., 2015; DiNapoli et al., 2016). These context-specific process improvements contribute to patient safety but may not show in measurements on guideline adherence. An in-depth description could help understand how ICLN’ workarounds, adaptions and adjustments to protocols contribute to safe practice. It might reveal possibilities to further reduce the gap between infection prevention policies (work-as-imagined) and their application in the variety of local contexts within the hospital environment (work-as-done) (Patriarca et al., 2020).

Our study findings should be interpreted in light of some limitations. The project leader of the link nurse program in our hospital is also the main researcher, which might have introduced social desirability bias. However, the link nurses from the hospital of the project leader were interviewed by an independent researcher (BS). Also, we did not see differences in the answers from the interviews with link nurse from other hospitals. Second, as link nurses volunteered to participate in the interviews, this increased the risk of including only highly motivated respondents. The responders in our interviews, however, mentioned both positive and negative experiences with the link nurse role and program; this makes such a bias less likely. A third limitation is that we performed the focus group interviews through an online platform and experienced some technical difficulties. We did not experience restrictions in interpersonal exchanges and encouraged interaction; nevertheless it could have limited the interaction between participants.

A strength of his study is the multisite design, resulting in a diverse sample of link nurses in different working environments (e.g. hospital and ward) and the variety in years of experience as a nurse and as an ICLN. It provided the possibility to explore the experiences of ICLN in various settings. The qualitative design added to the depth of the information and provided descriptions of their implementation efforts in everyday practice.

In conclusion, this analysis of experiences and perceptions of ICLN points to the importance of inspiration and support to help ICLN in assuming their role. With these preconditions in place, ICLN are more likely to feel empowered and consequently more likely to take impactful initiatives that contribute to the uptake of safe practices at the ward level. Therefore, activities to improve resilience and the empowerment of ICLN should be one of the pillars of ICLN programs.
